# Targeting Angiopoietin in Retinal Vascular Diseases: A Literature Review and Summary of Clinical Trials Involving Faricimab

**DOI:** 10.3390/cells9081869

**Published:** 2020-08-10

**Authors:** Majid Khan, Aamir A. Aziz, Noah A. Shafi, Tayeb Abbas, Arshad M. Khanani

**Affiliations:** 1 Reno School of Medicine, University of Nevada, Reno, NV 89557, USA; majidk@med.unr.edu (M.K.); nshafi@med.unr.edu (N.A.S.); tabbas@med.unr.edu (T.A.); 2 Sierra Eye Associates, Reno, NV 89502, USA; aamir.aziz@nevada.unr.edu

**Keywords:** VEGF-A, Ang-2 and Ang-1 pathway, Tie-2 receptor, diabetic eye disease, VE-PTP, vascularization, VEGF, diabetic macular edema, retinal vascular disease, neovascular age-related macular degeneration

## Abstract

This review summarizes the latest findings in the literature of Angiopoietin-2 (Ang-2), Tyrosine-protein kinase receptor (Tie-2) complex, and faricimab along with their involvement for the treatment of retinal vascular diseases in various clinical trials. In ischemic diseases, such as diabetic retinopathy, Ang-2 is upregulated, deactivating Tie-2, resulting in vascular leakage, pericyte loss, and inflammation. Recombinant Angiopeotin-1 (Ang-1), Ang-2-blocking molecules, and inhibitors of vascular endothelial protein tyrosine phosphatase (VE-PTP) decrease inflammation-associated vascular leakage, showing therapeutic effects in diabetes, atherosclerosis, and ocular neovascular diseases. In addition, novel studies show that angiopoietin-like proteins may play an important role in cellular metabolism leading to retinal vascular diseases. Current therapeutic focus combines Ang-Tie targeted drugs with other anti-angiogenic or immune therapies. Clinical studies have identified faricimab, a novel bispecific antibody designed for intravitreal use, to simultaneously bind and neutralize Ang-2 and VEGF-A for treatment of diabetic eye disease. By targeting both Ang-2 and vascular endothelial growth factor-A (VEGF-A), faricimab displays an improved and sustained efficacy over longer treatment intervals, delivering superior vision outcomes for patients with diabetic macular edema and reducing the treatment burden for patients with neovascular age-related macular degeneration and diabetic macular edema. Phase 2 results have produced promising outcomes with regard to efficacy and durability. Faricimab is currently being evaluated in global Phase 3 studies.

## 1. Introduction

Retinal vascular disease (RVD) understanding and treatment has increased tremendously over the last decade; moreover, there is an increased focus on improving the current anti-angiogenesis mechanisms and agents. Different disease mechanisms exist perpetuating a variety of different diseases; commonly, these include diabetic macular edema (DME), “wet” age-related macular degeneration (nAMD), and central or branch retinal vein occlusion (CRVO/BRVO, respectively). These disease processes are very prevalent. For instance, diabetes mellitus (Type I or Type II) is estimated to increase by 56% in the U.S. by 2030 [1], with DME causing visual imparity in up to 25% of patients [2]. Given the current therapeutic advancements, there is a strong need for reducing treatment burden and improving efficacy. Currently, patients with diabetic macular edema receive monthly or bimonthly intravitreal (IVT) injections in a clinical setting. Consequently, new molecules are designed to improve efficacy and durability compared to the current standard of care.

Diabetic retinopathy pathogenesis is well understood to occur due to the elevated levels of vascular endothelial growth factor (VEGF) resulting from retinal ischemia. VEGF is present in four isoforms (VEGF-A, -B, -C, and -D), which signal through three transmembrane tyrosine kinase receptors (VEGFR-1, -2, and -3) [3]. These VEGF associated regulators of disease are key therapeutic targets for the development of treatments for retinal pathologies involving angiogenesis (i.e., DME and nAMD). Current widely-used intravitreal agents target VEGF-A to suppress the abnormal growth of blood vessels and show a reduction in disease progression. 

The emergence of anti-VEGF agents has revolutionized the treatment of retinal vascular diseases. Two significant, commonly utilized anti-VEGF agents approved by the Food and Drug Administration (FDA) for treatment of DME include aflibercept and ranibizumab. Aflibercept is a 115 kDa soluble, recombinant protein that fuses the second extracellular domain of human VEGFR-1 and the third extracellular domain of human VEGFR-2 with the Fc component of human IgG [4]. The mechanism of action of aflibercept is to competitively inhibit the binding of VEGF to its corresponding receptors, VEGFR-1 and VEGFR-2 [5]. Studies indicate that aflibercept has a higher binding affinity for all VEGF isoforms, including VEGF-B, compared with the native receptors, and also binds placental growth factor (PlGF). These structural characteristics enable aflibercept to act over an extended period to suppress neovascularization and vascular permeability induced by VEGF overexpression [6]. Ranibizumab is a 48 kDa recombinant, humanized Fab fragment with the Fc component stripped away from the parent molecule. The mechanism of action of ranibizumab is high affinity binding to VEGF-A and subsequent inhibition of all isoforms of VEGF-A [7]. The literature supports that ranibizumab exhibits increased affinity for all isoforms of the VEGF-A molecules, rapid systemic clearance, and high systemic safety [4].

An emerging pathway utilizing the Angiopoietin-Tyrosine-protein kinase (Ang-Tie) system has been identified as a significant area for clinical drug development for retinal diseases. This pathway-axis plays an important and complementary role alongside VEGF vessel formation, though only Angiopoietin-1 (Ang-1) is involved in vessel maturation and intravesical inflammation prevention. The current therapeutic focus combines anti-Ang-Tie drugs with other anti-angiogenic (i.e., anti-VEGF-A) or immune therapies. Vascular endothelial protein tyrosine phosphatase (VE-PTP), a negative regulator of Tie-2, is also a significant therapeutic target. Recombinant Ang-1-, and Ang-2-blocking molecules, including inhibitors of VE-PTP, function in decreasing inflammation-associated vascular leakage and have therapeutic effects in diabetes, atherosclerosis, and ocular neovascular diseases.

This review summarizes the latest findings in the literature of VEGF, angiopoietin and angiopoietin-like factors, and the Ang-2/Tie-2 complex. In addition, an overview of faricimab and its uses for the treatment of retinal vascular diseases in various clinical trials is provided, which includes a summary of past study data and presentation of study designs in ongoing clinical studies.

## 2. Materials and Methods

### 2.1. Search Strategy

To investigate for previous reports of Ang-2 and Tie-2 in the literature, we performed a search using PubMed/MEDLINE to locate articles published on the topic, including literature reviews, case reports and series, clinical and observational studies, and meta-analyses with a specific aim in identifying articles pertaining to diabetic eye diseases. We first searched the database using the keywords “Ang-2, Ang-1, Tie-2 pathway,” yielding 31 results. While applying article-type filters and including keywords “VE-PTP AND VEGF,” this search yielded a total of 17 articles. A separate search for “ANG-2 receptor AND diabetic eye disease,” yielded 28 results. A different search of the database using the keywords “Angiopoietin 2 AND inflammation” yielded 77 results while applying a publication date filter of 5 years. Following, we searched the database using the keywords “angiopoietin-like protein AND diabetic retinopathy,” yielding 31 results. Afterward, the authors entered “extracellular vesicles AND angiopoietins,” yielding 30 results. Lastly, we searched for review articles using “Therapeutic Potentials AND Retina OR Gene therapy AND Port delivery system,” yielding 16 results. Correspondingly, following the searches, references were also identified from within respective article references.

### 2.2. Exclusion and Inclusion Criteria

The articles selected involved experimental (i.e., in vitro) and clinical research on humans and animals, or literature reviews and meta-analyses, published in the English language. Further, letters, case reports, and Ph.D. theses were excluded. Research classification included completed or ongoing prospective clinical trial data.

## 3. Angiopoietin Biology and Signaling, Literature Review

### 3.1. Overview of the VEGF Pathway-Axis

Discovery of the VEGF pathway in the treatment of diseases, such as diabetic retinopathy and nAMD, has facilitated many treatments that are currently being used as the standard-of-care. These therapies have been found to not only inhibit vasculogenesis but also the formation of tumors [8]. In DME patients, VEGF levels are upregulated, further worsening disease progression. Hence, targeting VEGF (subsequently blocking its action) or employing anti-VEGF therapies has proved to be effective [3]. The various isoforms of VEGF (-A, -B, -C, and -D) have different implications: VEGF-A: a growth factor that stimulates migration and replication of endothelial cells; VEGF-B: a vascular growth factor that acts as an antioxidant and downregulates pathological angiogenesis [9]; VEGF-C: a lymphatic vessel growth factor (promoting lymphangiogenesis) [10]; and VEGF-D: a ligand for lymphatic growth factor receptor VEGFR-3/Flt-4 that can also act as a malignancy biomarker (i.e., sporadic lymphangioleiomyomatosis) [11]. Targeting these different isoforms of VEGF (VEGF-A, -B, -C, and -D) and their respective transmembrane tyrosine kinase receptors (VEGFR-1, -2, and -3) has been the forefront of many pharmacological studies. Inactivation of the receptors can prevent further progression of many isoforms in the VEGF pathway. Other inflammatory markers are also noted to be upregulated in the aqueous humor of patients with DME, such as interleukin-6 (IL-6), a cytokine with a role in pro-inflammatory immune responses that is seen to be positively correlated with VEGF [12]. Early stage trials are looking at the efficacy and safety of IL-6 inhibition in patients with DME. Plasma kallikrein inhibition and integrin antagonists have also shown promising data in Phase 1 studies [13,14]. Recent Phase 1B/2A trials utilizing VEGF-C and VEGF-D inhibition via OPT-302 in patients with DME also met the primary endpoint [15]. Although inhibition of multiple different pathways has shown potential benefit in early clinical trials, VEGF-A remains one of the primary targets for new retinal treatments, but its interaction with the novel Ang/Tie pathway-axis may provide new insight for the treatment of retinal diseases, such as nAMD and DME. 

### 3.2. VEGF and Ang-2 in Diabetic Eye Disease

Co-expression of Ang-2 and VEGF-A has been reported to produce accelerated neovascularization in the developing retina and ischemic retina models. Moreover, Ang-2 and VEGF levels have been reported elevated in vitreous samples from diabetic patients [16]. Levels of serum VEGF and Ang-2 are also found more elevated in proliferative diabetic retinopathy groups than in the non-proliferative diabetic retinopathy groups, suggesting that their levels may be related to the progression of retinopathy [17]. These findings indicate the potential involvement of Ang-2 and VEGF in vascular permeability and pathological neovascularization.

### 3.3. Ang-2 Effects on Vasculature

#### 3.3.1. Angiopoietin Interactions

It has been found that the tyrosine kinase with immunoglobulin-like and epidermal growth factors-like domains 2 (EGF-like domain 2) pathway, Ang-1, and Ang-2 interact with one another to influence angiogenesis. Tie-2 is a transmembrane receptor that is selectively found on the endothelial cells of blood vessels and functions as a binding site for Ang-1 and Ang-2. However, Ang-1 and Ang-2 compete with one another when binding to Tie-2. In addition, binding of either Ang-1 or Ang-2 leads to different effects on the Tie-2 pathway. Studies show that, when Ang-1 binds to Tie-2, causing phosphorylation of the receptor and initiating its effects of inhibiting vascular permeability, it also preserves vascular stability [18,19]. While Ang-1 is a full agonist of Tie-2, Ang-2 can function as a partial agonist or antagonist. Furthermore, binding of Ang-2 to Tie-2 inhibits phosphorylation, thereby deactivating the entire pathway [19,20].

#### 3.3.2. Utilizing Ang-Signaling for Favorable Vascular Outcomes

Analogous in function to the vascular-stabilizing function of Ang-1, recombinant Ang-1 protein has demonstrated inhibition of vascular leakage induced by inflammatory cytokines and growth factors. Recent studies utilizing injected recombinant Ang proteins suggest that Ang-1 protects against diabetic vascular dysfunction. Notable findings include reduced capillary vasoregression, vascular permeability, and retinal hypoxia. These changes improved neurovascular prognosis, neural dysfunction (particularly in diabetic retinopathy), and vision [21]. Therapeutic applications of Ang-1 are present in malignancies where vascular protection by Ang-1 is suppressed, and when endothelial dysfunction and vascular disease are predisposed.

Under conditions of hyperglycemia, hypoxia, or oxidative stress, levels of Ang-2 are upregulated. Reversal of Ang-1 and Ang-2 expression has been shown in malignancies. Thus increases in serum Ang-2 levels offer prognostic value for certain cancers and have been correlated with tumor progression, angiogenesis, metastasis, and patient survival [16]. Diabetic mouse models have supported that hyperglycemia also results in increased Ang-2 levels and expression of integrin α3 and β1 in pericytes, leading to pericyte apoptosis via the p53 pathway. Intravitreal injection of anti-integrin α3 and β1 antibodies lessened this Ang-2 induced pericyte loss [22]. Many drugs have been designed to inhibit Ang-2, thereby allowing more Ang-1 to activate the Tie-2 receptor and culminating in anti-permeability effects.

### 3.4. Regulation of the Inflammatory Processes by Angiopoietins

Inflammation and angiogenesis are established as highly interconnected processes with many factors identified with dual function in both of these significant pathways. In physiologic conditions, activated Tie-2 maintains the endothelium in the quiescent state marked by dynamic barrier function and anti-adhesion against leukocytes in circulation [23]. In diseased states, however, a marked imbalance in the expression of angiopoietins develops, thereby attenuating Tie-2 signaling [23]. These rapid molecular changes enhance systemic pathophysiologic responses, such as injurious vascular leakage and organ inflammation.

#### 3.4.1. Ang-1

The action of Ang-1 in counteracting vascular hyperpermeability is induced by TNF-*α* alongside other cytokines, Anthrax lethal factor, thrombin, as well as many other triggers [24]. In consideration of the impressive defense against unrelated ligands acting on the endothelium, it is supported that Ang-1 (via signaling to conserved downstream regulators) acquires endothelial intercellular connectivity and cell shape vascular barrier defense [24,25].

Angiopoietin-1 (Ang-1) functions in reorganizing the actin-myosin cytoskeleton of the endothelial cell. This manifests as the spread of borders toward the periphery of the cell and the development of tensile strength at the cells’ edges. When activated, Tie-2 migrates to the cell junction, where trans-interactions via Ang-1 provide increased strength to intercellular connections. In addition, vascular endothelial cadherin (VE-cadherin), an adherens junction protein, proves vital for barrier defense against inflammatory stressors [26]. This is owing to the fact that Ang-1 stabilizes VE-cadherin at the adherens junction [27]. In the ways addressed, Ang-1 notably reduces vascular inflammation, thus lessening the entry of immune cells into the parenchyma.

#### 3.4.2. Ang-2

As previously mentioned, Ang-1 is an agonist of Tie-2, whereas Ang-2 is a partial agonist or antagonist of Tie-2. Early studies support that Ang-2 competitively inhibits Ang-1–induced Tie-2 activation when in the context of endothelium [28]. Across different disease models, it is supported that Ang-2 is depleted by antibodies, deleted genetically, or knocked down by ribonucleic acid (RNA) interference. Each of these manifestations of loss-of-function presents with the same outcome —reduced vascular leakage, improved organ function, and increased survival in pathological conditions [24]. Due to the fact that Ang-2 inhibition parallels the findings of Ang-1 in excess—even in measures of Tie-2 activation—these outcomes support the indicated role of Ang-2 as an antagonist of Tie-2 in systemic inflammation.

When exposed to inflammatory cytokines, endothelial cells respond by attenuating the intercellular connections and positioning of inflammatory adhesion molecules on the cell surface. This enables leukocyte exit from circulation and entry into tissues and release of preformed Ang-2 protein from Weibel–Palade bodies [25]. Ang-2 amplifies the cytokine-induced effects on the vasculature. This is notably supported by gene deletion models of sterile inflammation and bacterial sepsis [24]. 

Notably, Ang-2 protein functions in potentiating leakage and adhesion responses of blood vessels in response to cytokines. Ang-2 also reinforces its own production via Forkhead Box O1 (FOXO1) [29]. In quiescent endothelium, a signal transduced by activated Tie-2 inhibits Foxo1 from transcribing the Ang-2 gene. Consequently, lower levels of Ang-2 are produced and stored in Weibel–Palade bodies. In contrast, when exposed to inflammatory stimuli, there is a rapid release of preformed Ang-2 protein—this is thought to antagonize Tie-2, leaving FOXO1 uninhibited. Free, activated, FOXO1 inflamed endothelial cells produce Ang-2 protein, which in turn promotes Tie-2 inhibition, further increasing Ang-2 gene transcription [30]. By way of Ang-2 interactions with FOXO1, an aberrant positive feedback loop sets in motion the development of systemic inflammation.

### 3.5. Vascular and Extravascular Functions of Angiopoietin-2

Angiopoietin-2 (Ang-2) serves many significant functions across vascular and extravascular settings. This is showcased well in disease conditions, such as hyperglycemia, hypoxia, or oxidative stress, where Ang-2 is markedly upregulated [16]. Diabetic macular edema (DME) and diabetic retinopathy, among other pathologic states, exemplifies the role of Ang-2 in stimulating pericyte loss, neovascularization, astrocyte loss in Blood-Brain Barrier (BBB), and breakdown of the Blood Retinal Barrier (BRB). 

#### 3.5.1. Pericyte Loss 

Retinal vasculature contains the highest pericyte coverage observed in the body. The high 1:1 ratio of pericytes to endothelial cells in the retina is important to the organ’s function in vascular stabilization, specifically in the regulation of the endothelium, and control of blood flow and capillary permeability. Since the retinal vasculature happens to be the most sensitive site for pericyte loss, this is among the first morphological changes observed in the diabetic retina [31]. The role of Ang-2 in stimulating Tie-2 phosphorylation in pericytes and the mechanism of pericyte migration due to disease conditions are still poorly understood. However, studies in diabetic mouse models support that Ang-2 induces pericyte migration through the Tie-2 receptor, specifically the Ras-dependent extracellular signal-regulated kinase (ERK1/2) and serine and threonine kinase/protein kinase B (Akt/PKB) pathways. ERK1/2, the dominant pathway, suppresses other pathway activities and increases pericyte migration [32]. While these findings are promising, further studies are required to characterize this mechanism better.

#### 3.5.2. Neovascularization

Conditions of hypoxia and angiogenesis in the retina increase the expression of Ang-2. In conjunction with VEGF expression, Ang-2 facilitates the process of angiogenesis. This occurs through interaction between Ang-2 and Tie-2, which produces disturbances in endothelial junctional integrity, pericyte loss, and a general priming of the vascular bed for angiogenic sprouting [25]. In studies of rat models, a 30-fold increase in Ang-2 is observed in the retinas of diabetic rats compared to those of nondiabetic rats [33]. In the context of the diabetic retina, such elevations in Ang-2 are conducive to angiogenesis and ultimately neovascularization. 

#### 3.5.3. Astrocyte Loss Compromise the Blood-Brain Barrier

Diabetic retinopathy produces vascular leakage that results in macular edema and vision loss. Astrocytes play a significant role in the regulation of BBB integrity in the brain. Furthermore, rat models of diabetic retinopathy demonstrate that as Ang-2 expression is increased, astrocyte loss occurs and vascular leakage results [34]. A similar study in rat models with elevated expression of Ang-2 showed notable decreases in adherens junctions, reduced pericyte coverage, disrupted glycocalyx, and increased caveolin-1 (a vesicular-permeability related molecule) [35]. These findings back the role of Ang-2 in mediating permeability and disruption of the BBB through both paracellular and transcellular routes. 

#### 3.5.4. Breakdown of the Brain Retinal Barrier

In physiologic conditions, BRB integrity is maintained by specialized intercellular junctional molecules and adhesive interactions between endothelial cells, alongside their associated pericytes [34]. Early in the onset of diabetic retinopathy, there is an alteration of the BRB that produces increased permeability of the retina through dysregulation of the aforementioned junctions, finally culminating in DME. Diabetic rat models demonstrate the role of Ang-2 in inducing morphological changes in the endothelial monolayer. Specifically, elevations in Ang-2 expression show a decrease in VE-cadherin function and increase space lining the endothelial monolayer [36]. Further study of these morphological changes will prove to be valuable in expanding our understanding of the role of Ang-2 in DME and other pathologic conditions. 

### 3.6. Regulation of Cell Metabolism by Angiopoietins

#### 3.6.1. Angiopoietins and Glucose

Angiopoietins are growth factors that play a crucial role in vascularization. Aside from influencing blood vessel growth and inflammation via the Tie pathway, the angiopoietin superfamily are also regulators in cell metabolism. It is known that chronic conditions, such as Type 2 diabetes, occur when there is insulin resistance causing decreased glucose absorption into cells, which results in increased glucose levels in the bloodstream. Consequently, excess blood sugar levels eventually trigger microvascular lesions in the retina, which can lead to diabetic retinopathy (i.e., DME) and eventual permanent vision loss [37]. Because Type 2 diabetes and cell metabolism are crucial components of diabetic retinopathy development, many studies have investigated whether angiopoietins are a part of glucose and fat metabolism.

Angiopoietin-like proteins (ANGPTL) are similar in genotype and structure to the original angiopoietins. Santulli et al. [38] report an observed difference between the two is their function; moreover, while angiopoietins, such as Ang-1 and Ang-2, interact with Tie-2 and regulate blood vessel formation, ANGPTL has distinct roles in glucose and lipid metabolism. For instance, with regard to insulin as a key modulator of ANGPTL-3, more research is warranted to elucidate a concrete understanding. Many studies have shown that ANGPTL-3 concentrations are increased in patients with Type 2 diabetes when compared to those without diabetes [39]. Although the specific mechanism is unclear, some researchers speculate that insulin resistance could be a result of increased fatty acid circulation secondary to increased lipolysis [40]. On the other hand, researchers believe that ANGPTL-3 decreases triglyceride formation, which causes low insulin sensitivity [41]. 

#### 3.6.2. Angiopoietin-Like Proteins and Disease Processes

ANGPTL-4 is another angiopoietin-like protein that influences cell metabolism. However, data suggests that ANGPTL-4 has different effects depending on whether test subjects are obese, have Type 2 diabetes, or have overexpression of ANGPTL-4. To further illustrate this data, one study found that subjects who were morbidly obese had higher concentrations of ANGPTL-4 compared to those who were not obese and nondiabetics [42,43]. In addition, a study by Liechtenstein et al. [44] showed insulin resistance was associated with overexpression of ANGPTL-4, which further suggests that ANGPTLs play a role in metabolic processes and even in the dysregulation of metabolic syndromes, such as diabetes.

Further, ANGPTL-8 is also believed to play a role in fatty acid metabolism. Just like the other angiopoietin-like proteins, ANGPTL-8 is highly regulated and is most active in the prandial state, which then increases the ANGPTL-8 and -3 pathway [45,46,47]. However, studies have shown that in mice models, concentrations of ANGPTL-8 affect triglyceride levels in a linear fashion [48]. Similar to ANGPTL-3, the mechanism is still under investigation, but there is promising research that suggests ANGPTL-3 and ANGPTL-8 work in conjunction with one another in the breakdown and transport of fats. 

It is well established that DME is the result of microaneurysms that occur in the blood vessels of the retina, leading to the accumulation of fluid within the macula. Research shows that chronically uncontrolled hyperglycemia further exacerbates retinopathy causing increased angiogenesis. Overall, ANGPTL has a critical role in glucose and fatty acid metabolism and trafficking throughout the cell. While further analysis of these proteins is still required, possible treatments may include medications that regulate ANGPTL activity. By controlling the cell’s energy metabolism, these therapeutics may downregulate pathologic neovascularization and further prevent retinopathy and macular edema.

### 3.7. Extracellular Microvesicles and Angiopoietin Biology

#### 3.7.1. Overview of Extracellular Microvesicles

Extracellular microvesicles (EVs) are lipid-encapsulated cellular particles of the cell membrane that interact with other cells as a means of communication. EVs are important for the growth of healthy tissues and serve as a final messenger during apoptosis [49]. While EVs are controlled in very specific manners under physiological conditions, it remains to be seen if EVs are as strictly regulated in pathological disease states, such as diabetic retinopathy (i.e., DME). In these chronic conditions, vascularization develops from pre-existing blood vessels that endure microtears within the cellular lining. Consequently, in the last decade, many studies in Type 2 diabetes mellitus, diabetic retinopathy have analyzed whether angiopoietins utilize EVs as a means of initiating angiogenesis [50]. 

#### 3.7.2. Extracellular Microvesicle Genetic Changes in Diabetics

Under normal conditions, angiogenesis is heavily monitored. Studies show that various proteins are enclosed in vesicles, some of which include VEGF, platelet-derived growth factor (PDGF), and fibroblast growth factor-2 (FGF-2) that act as stimulating factors for angiogenesis [51]. In addition, transcription factors like signal transducer and activator of transcription-3 and -5 (STAT-3 and STAT-5) also initiate vascularization when released in EVs [52,53,54]. However, more recent data suggest that differences in the miRNA sequence can lead to increased blood vessel growth, as well. A study by Mazzeo et al. [55] reveals that EVs of patients with diabetes contain specific miRNA patterns that were different when compared to EVs of a nondiabetic control group. In addition, from previous work, they showed that EVs of patients with diabetic retinopathy have properties that further stimulate diabetic retinopathy in vitro [56]. Although further investigation is needed, potential therapeutics could include targeting specific RNA sequences in patients with diabetic retinopathy to prevent increased vascular permeability of damaged blood vessels.

## 4. Clinical Studies of Faricimab in Diabetic Macular Edema

### 4.1. Therapeutic Potentials of Angiopoietins, Emerging Pathways

Efforts to improve the current treatment burden and efficacy include the development of extended-delivery type mechanisms and gene therapy. A pipeline of investigational drugs and mechanisms is expected to extend or eliminate the dosage requirements of currently available agents. These breakthroughs aim to utilize pathways beyond anti-VEGF, and in the future may take advantage of the Ang-2/Tie-2 complex (e.g., via faricimab) or individual angiopoietins (i.e., ANGPTL-3).

### 4.2. Overview of Faricimab

Within the last decade, an increase in understanding and advancements has allowed for better treatment of retinal diseases using anti-angiogenesis pathways and agents. Further, with an aim to improve the current standard-of-care treatment options, notably in patients suffering from nAMD and DME, a need for treatments and approaches utilizing new, or improving upon current, biological pathways and mechanisms is still evident. This need is especially evident when considering the current treatment burden of monthly IVT clinic appointments, extended-release type mechanisms are of high demand [57,58]. One molecule of interest, faricimab, aims to bridge this gap and is further discussed along with the clinical trials for application in the treatment of nAMD and DME patients. A summary table of these clinical trials is provided below (Table 1).

### 4.3. Faricimab for Treatment of Disease

With the biological implications of angiopoietin and approaches described, faricimab (formerly RG7716), is the first bispecific antibody designed for intravitreal use, and can simultaneously bind to and neutralize Ang-2 and VEGF-A for treatment of nAMD and DME [16,59,60]. Patients with retinal vascular diseases deemed anti-VEGF sub-responders by retina specialists demonstrate a lack of visual acuity improvement or persistent anatomical symptoms, such as intra- or subretinal fluid. The current standard of care molecules exclusively targets VEGF, creating an unmet need for anti-VEGF sub-responsive patients. Faricimab aims to improve these patients’ prognoses by providing an alternative biological pathway for their treatment by inhibiting both VEGF and Ang-2. By simultaneously binding to both targets, faricimab can provide improved efficacy and durability in patients with DME and durability in nAMD—inherently decreasing treatment burden [59,60], a targeted area of focus for researchers and ophthalmologists.

Initial studies showed that vitreous levels of Ang-2 are elevated in nAMD, diabetic retinopathy (i.e., DME), and retinal vein occlusion [19,61,62]. In animal studies, spontaneous choroidal neovascularization models show faricimab and other VEGF-A/Tie-2 targeting agents reduce vessel lesions and neuron loss, inhibit retinal leukocyte infiltration, and prolong the anti-leakage effect [62,63]. Preclinical studies showed that blocking Ang-2 reduces VEGF-induced endothelial barrier breakdown [62,64]. Further, a successful Phase 1 study in nAMD confirmed patient safety and tolerance, while simultaneously improving BCVA and anatomical parameters for patients [61]. 

Phase 2 studies were designed to further evaluate efficacy and safety of faricimab in patients with DME (BOULEVARD) and nAMD (AVENUE and STAIRWAY), evaluating safety and efficacy, along with comparing to the current standard-of-care, ranibizumab. Improvements in BCVA, central subfield thickness (CST), and diabetic retinopathy severity score (DRSS) were noted in patients with DME. Moreover, in nAMD, patients treated with faricimab every 16 weeks and every 12 weeks showed similar outcomes as monthly ranibizumab. Due to positive data from the Phase 2 trials, ongoing Phase 3 studies aim to further understand faricimab and its effects in DME (YOSEMITE and RHINE) and nAMD (TENAYA and LUCERNE). Data from these studies aim to look at the primary outcomes in mean BCVA at 1-year post-treatment and will support the future direction for its expanded use in treatment for the patients suffering from nAMD and DME. These studies are discussed below—initially in Phase 2, DME and nAMD, and later in Phase 3, DME, and nAMD, respectively.

### 4.4. Phase 2 BOULEVARD in DME

BOULEVARD was a prospective, Phase 2, randomized, active comparator-controlled, double-masked, multicenter study evaluating safety and efficacy in patients with DME [61,65]. The study recruited 229 patients (168 naïve and 61 previously-treated) at 59 clinical sites in the U.S. The trial included patients with BCVA from 73 to 24 early treatment diabetic retinopathy (ETDRS) letters and central subfield thickness of 325 microns or higher. Ranibizumab 0.3 mg (Lucentis; Genentech) was used as a comparator against two doses of faricimab, 1.5 mg or 6.0 mg. The primary endpoint of the study was mean change in BCVA at week 24 in treatment naïve patients. In the 6.0 mg group, statistically significant superior visual acuity gains (BCVA) were made with faricimab at week 24, with a mean gain of +13.9 letters from baseline and +3.6 letters more than ranibizumab. Outcomes also included dose-dependent improvement in CST reductions, DRSS improvements, and a longer time to retreatment during the observation period compared to ranibizumab, with no new or unexpected safety signals [66]. Many patients treated with faricimab displayed longer retreatment intervals, showcasing this novel antibody’s ability to reduce monthly DME standard-of-care.

### 4.5. Phase 2 AVENUE and STAIRWAY in nAMD

Two studies assessed the safety, efficacy, and extended-dosage of faricimab; the 273-patient AVENUE and 76-patient STAIRWAY studies, respectively [67]. AVENUE examined treatment naïve nAMD patients and consisted of 5 cohorts; a 0.5 mg ranibizumab control every four weeks, a 1.5 mg faricimab every four weeks, a 6.0 mg faricimab every four weeks, a 6.0 mg faricimab every eight weeks, and a 0.5 mg ranibizumab group receiving three monthly loading doses then switched to 6.0 mg faricimab every four weeks. All groups showed BCVA improvement and CST reductions.

Furthermore, the STAIRWAY study examined 6.0 mg faricimab at extended-dosing of 12- and 16-week intervals against 0.5 mg ranibizumab every four weeks for treatment naïve nAMD patients [64]. Three months after the last of four loading doses, patients randomized to faricimab were evaluated for disease activity. Patients with active disease were shortened to a 12-week faricimab cohort. The results showed that 65% of patients in the faricimab treated cohort had no disease activity at week 24, 12 weeks after the last dose. This study, conducted over 52 weeks, showed mean BCVA improvements for the 16-week faricimab arm (+11.4 letters), 12-week faricimab cohort (+10.1 letters), and the ranibizumab cohort (+9.6 letters). Comparable CST reductions were noted in all three groups, with no observed safety signal observations. The overall safety profile across all trials of faricimab showed consistent safety profiles, like other intravitreal anti-VEGF treatments, prompting Phase 3 study. 

### 4.6. Phase 3 YOSEMITE and RHINE in DME

Due to the positive data from BOULEVARD, the Phase 3 YOSEMITE and RHINE trials were designed to evaluate the safety, efficacy, and durability of faricimab in patients with DME [68]. YOSEMITE and RHINE are multicenter, randomized, double-masked studies with aflibercept as the active comparator. Each trial has enrolled over 900 patients each. Patients are randomized into three groups: one group receiving 6.0 mg faricimab every eight weeks and one group receiving 6.0 mg faricimab at personalized treatment intervals (PTI), while the control arm receives aflibercept every eight weeks, all after the monthly loading doses. Patients in all three arms will receive sham injections at various intervals to maintain masking. The primary outcome is the mean change in BCVA from baseline at one year. Secondary outcomes being evaluated include mean changes in BCVA from baseline up to two years, participants with a 2-step improvement in the DRSS, and changes in the CST from baseline up to two years. The studies are fully enrolled ahead of schedule, with data expected in the first quarter of 2021.

### 4.7. Phase 3 TENAYA and LUCERNE in nAMD

Phase 3 studies, TENAYA and LUCERNE, were initiated in over 1200 patients in nAMD globally [68,69]. TENAYA AND LUCERNE are multicenter, randomized double-masked studies, initiated in 2019, aiming to evaluate faricimab safety, efficacy, and durability against aflibercept for the treatment of nAMD patients. Patients will be randomized to either faricimab every 16 weeks flex group or aflibercept every eight weeks, with patients in the 16-week faricimab flex dropping to 12 weeks, or further to 8 weeks, as needed based on disease activity. Sham (e.g., placebo) injections are performed to conserve masking of the groups. The primary endpoint is the evaluation in the change of BCVA at week 48 from baseline, and preliminary results from these studies are anticipated in the first half of 2021.

## 5. Conclusions

Alongside VEGF, the Ang-2 Tie-2 pathway has been implicated in the pathogenesis of retinal vascular diseases, including pericyte loss, vascular leakage, and inflammation. These disease processes are linked with Ang-2 interaction with the Tie-2 receptor and downstream signaling pathways. As a result, astrocyte loss in the BBB and breakdown of the BRB are observed. Notably, in the presence of VEGF expression with Ang-2, angiogenesis and neovascularization are observed, as well. It is also worth mentioning that Angiopoietin-like proteins can potentially exacerbate pathophysiological symptoms of metabolic syndrome and worsen ocular diseases secondary to diabetes. The blocking Ang-2, in addition to VEGF-A, with faricimab results in Tie-2 activation and vascular stabilization. This may result in better visual acuity gains and durability of treatment in patients with DME, compared to standard-of-care, and better durability in patients with nAMD. Ongoing Phase 3 clinical trials will confirm the safety and efficacy of faricimab in patients with DME, as well as nAMD.

## Figures and Tables

**Table 1:** A summary of recent clinical trials involving faricimab.

Name	Disease	Basic Design	Results
**BOULEVARD**	DME	Faricimab (1.5 mg, 6.0 mg) vs. Ranibizumab 0.3 mg	Faricimab 6.0 mg was superior to Ranibizumab 0.3 mg in terms of BCVA and CST improvements at week 24.
**AVENUE**	nAMD	Faricimab (1.5 mg Q4W, 6.0 mg Q4W, 6.0 mg Q8W) vs. Ranibizumab (0.5 mg Q4W) vs. Ranibizumab (0.5 mg × 3 monthly doses switched to faricimab 6.0 mg Q4W)	All groups showed BCVA and CST improvements.
**STAIRWAY**	nAMD	Faricimab (6.0 mg Q12W, Q16W) vs. Ranibizumab (0.5 mg Q4W)	Faricimab Q16W and Q12W showed comparable BCVA and CST outcomes as monthly Ranibizumab 0.5 mg Q4W.
**YOSEMITE**	DME	Faricimab (6.0 mg Q8W, PTI) vs. Aflibercept (2.0 mg Q8W)	Trial currently ongoing.
**RHINE**	DME	Faricimab (6.0 mg Q8W, PTI) vs. Aflibercept (2.0 mg Q8W)	Trial currently ongoing.
**TENAYA**	nAMD	(Faricimab 6.0 mg Q16W Flex) vs. Aflibercept (2.0 mg Q8W)	Trial currently ongoing.
**LUCERNE**	nAMD	(Faricimab 6.0 mg Q16W) vs. Aflibercept (2.0 mg Q8W)	Trial currently ongoing.
